# Can cow’s milk allergic children on an elimination diet have a nutritional intake comparable to non-allergic children?

**DOI:** 10.1186/2045-7022-3-S3-P133

**Published:** 2013-07-25

**Authors:** A Toniolo, C Zorzin, MA Muraro, R Bonaguro, F Lazzarotto

**Affiliations:** 1 Department of Pediatrics - University of Padua, Food Allergy Centre – Veneto Region, Padua, Italy

## Background

The aim of our study was to compare the intake of nutrients and nutritional status of cow’s milk allergic (CMA) children supported by a dietician and children with no dietary limitations (NDL).

## Methods

We observed 54 children (mean age 8,2±4,5) with CMA which had at least one dietetic evaluation and received dietary support to adequately integrate their diet, and 54 children (mean age 8,4±4,7) with no dietary restrictions and no dietary intervention. The assessment of nutrient intakes (energy, proteins, lipids, carbohydrates, fibre, calcium, iron) was based on a 7-day food diary. All values were compared to the Italian Recommended Daily Allowance (RDA). Children were divided by age groups: 1-5years (1), 6-10years (2), 11-18years (3). Nutritional status was assessed by measuring weight and height related to the standard for age and sex.

## Results

The mean weight and height were within the norm range in both populations. 32% of NDL children and 23% of CMA had a mean weight-for-age percentile >75. In all age groups, CMA patients had a medium energy intake (EI) higher than the controls. The mean protein intakes of both populations at different ages were higher than the RDA. Mean carbohydrates (CHO) intakes were lower in healthy children than in children with CMA and there was no difference in mean fat intakes. The mean intake of calcium, iron and fibre were below the RDA in both populations (Table 1).

**Table 1:** 

	EI (kcal)	PROTEIN g (%)	CHO g (%)	FAT g (%)
**CMA;** **NDL (1)**	1217,4; 1100,4	42,9 (13,6); 40,3 (14,2)	174,0 (56,8); 149,8 (51,7)	41,4 (29,8); 42,4 (34,6)

**CMA;** **NDL (2)**	1776,7; 1759,5	57,5 (18,8); 60,1 (13,7)	252,8 (55,8); 226,3 (50,9)	64,0 (31,4); 71,0 (35,9)

**CMA;** **NDL (3)**	2131,7; 1920,6	69,1 (12,7); 67,9 (13,9)	297,8 (55,1); 249,8 (45,4)	77,6 (32,2); 75,6 (34,9)

## Conclusion

Our data show that children with CMA on an elimination diet, who received dietetic support, have a nutrient intake similar to children with no dietary limitations. The low calcium quantity in CMA children is a critical issue that should be monitored in other to supplement children, especially in the older groups (group 2 and group 3) with a higher RDA. These preliminary data confirm the importance of a dietetic support in allergic children in all age groups. The nutrient intake suggests that, also in children with no dietary restrictions, more attention should be paid to the nutritional aspects by paediatricians.

## Disclosure of interest

None declared.

